# Supplementary figures and images for: Brain tissue transcriptomic analysis of SIV-infected macaques identifies several altered metabolic pathways linked to neuropathogenesis and poly (ADP-ribose) polymerases (PARPs) as potential therapeutic targets
Source: J Neurovirol. 2021 Jan 6;27(1):101–15. doi: 10.1007/s13365-020-00927-z (PMC7786889; doi:10.1007/s13365-020-00927-z)

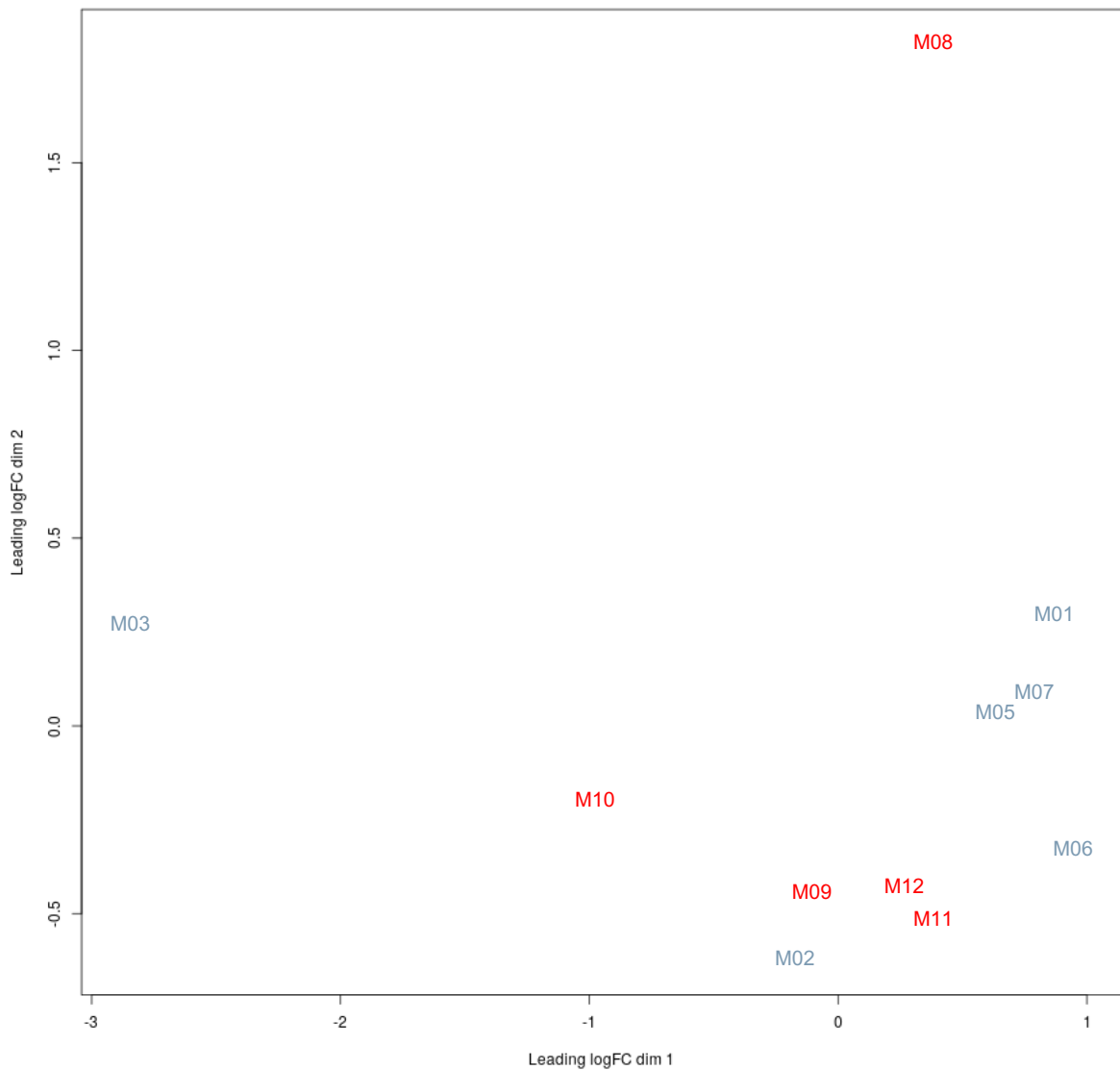

Supplement: Supplementary file 1 — Supplementary file1 (PDF 33 KB) [file 13365_2020_927_MOESM1_ESM.pdf]
